# Supplementary material for: Carbon Amendments Shape the Bacterial Community Structure in Salinized Farmland Soil
Source: Microbiol Spectr. 2023 Jan 10;11(1):e01012-22. doi: 10.1128/spectrum.01012-22 (PMC9927309; doi:10.1128/spectrum.01012-22)
Supplement: Supplemental file 1 — Supplemental material. Download spectrum.01012-22-s0001.pdf, PDF file, 0.3 MB [file spectrum.01012-22-s0001.pdf]

Table S1 Physicochemical properties of soil collected from Xinjiang Province

Total Nitrogen (TN, mg/g)	Soil organic matter (SOM, %)	Total phosphorus (TP, mg/g)	EC (ms/cm)	pH
0.41±0.03	0.75±0.07	0.46±0.01	4.11±0.06	8.07±0.16

Table S2 Other elemental analysis results for the biochar.

	Sample 1	Sample 2	Sample 3	Method
Cation exchange capacity (cmol(+)/kg)	24.1	24.0	23.1	Ammonium acetate exchange
Chloride ions (g/kg)	0.034	0.034	0.034	
Magnesium (g/kg)	1.2	1.3	1.2	
Calcium content (g/kg)	0.0	0.0	0.0	
Sodium content (g/kg)	0.5	0.4	0.5	Atomic absorption spectrophotometry
Iron content (g/kg)	0.26	0.27	0.26	
Copper content (g/kg)	8	8	8	
Lead content (g/kg)	5.4	4.7	5.7	
Arsenic content (g/kg)	1.23	1.51	1.47	
Zinc content (g/kg)	63	65	62	Atomic fluorescence spectrophotometer

Table S3 Hoagland solution with salt content (10L)

Component	content
MES	5g
$Ca(NO_3)_2 4H_2O$	20ml
KNO_3	20 ml
NH_4NO_3	10 ml
$MgSO_4 7H_2O$	20 ml
KH_2PO_4	10 ml
KCL	2 ml
Micro elements	5 ml
Fe-EDTA	40 ml

Salt concetration		
Salt concentration	NaCl (g)/10L	$CaCl_2$ (g)/10L
50 mM	29.400	27.750

Table S4 The networks of key topological features in the compost bacterial community of different treatments and type soils.

	CK	DA	BA	DBA	BS	RS	SS
Total nodes	267	316	298	272	297	348	365
Edge	1770	3878	2512	1763	2635	4642	5554
Average_degree	13.25843	24.5443	16.85906	12.96324	17.74411	26.67816	30.43288
Average_path_length	4.382159	3.762584	3.761323	5.001012	4.729876	3.055122	3.487205
Network_diameter	15	11	14	11	18	9	12
Clustering_coefficient	0.562326	0.573223	0.571542	0.522077	0.538977	0.539138	0.544833
Density	0.049844	0.077918	0.056765	0.047835	0.059946	0.076882	0.083607
Heterogeneity	1.265931	1.118902	1.161665	1.003149	1.258635	0.828607	0.833495
Centralization	0.205795	0.21732	0.212596	0.162497	0.264378	0.188247	0.207602

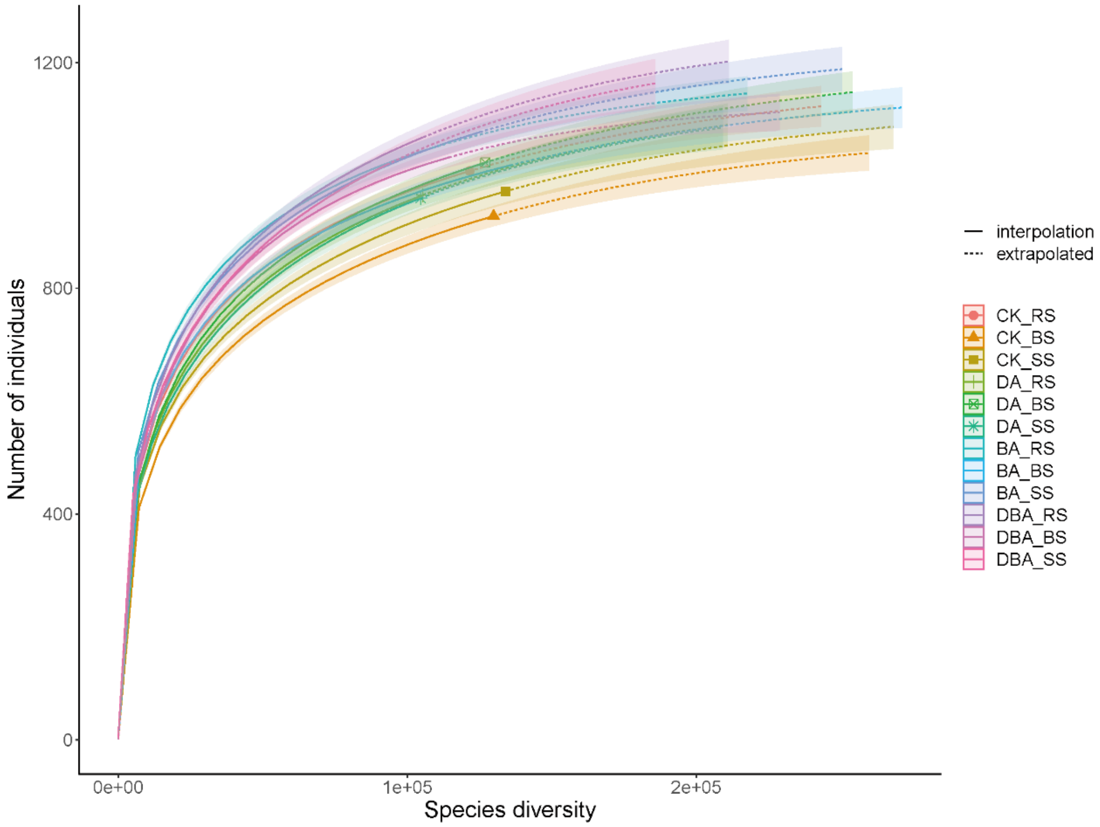

Figure S1 The samples of the species accumulation curve for different treatments.

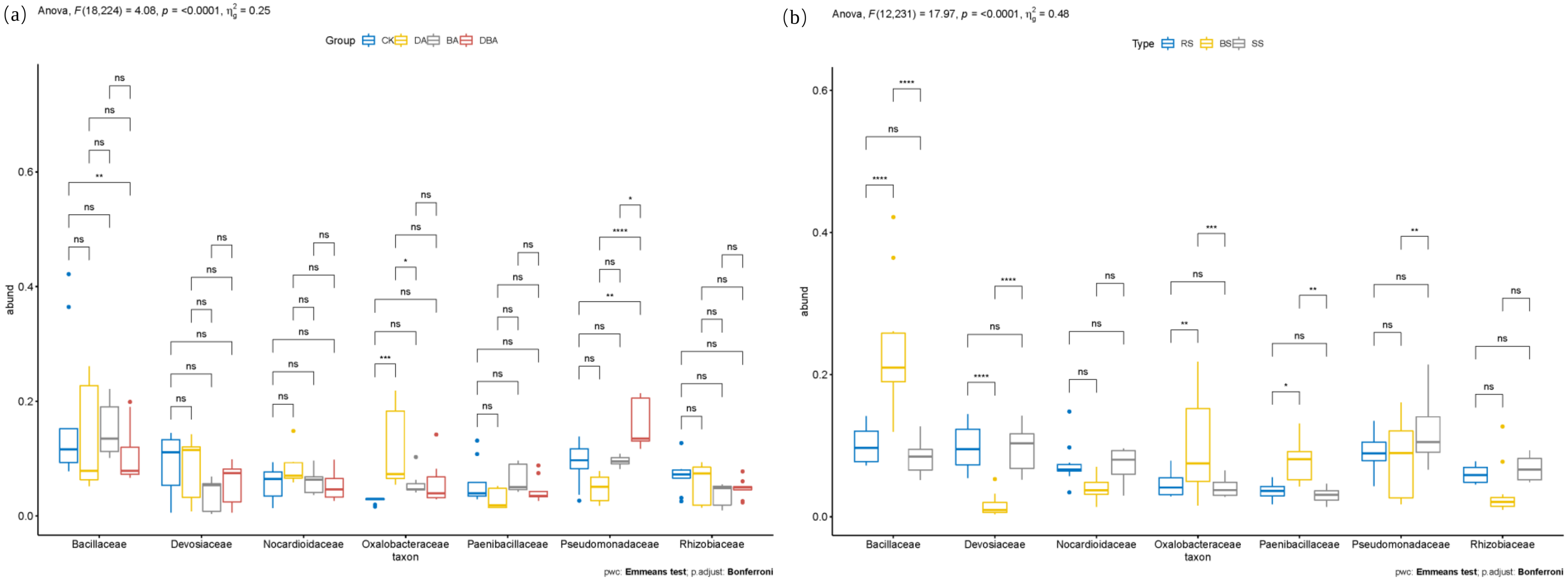

Figure S2 Boxplot showed the difference in the relative abundances of the top seven families. (a) four treatment (b) three soil types. *, significantly (p< 0.05), ** significantly (p< 0.01), * significantly (p< 0.001), **** significantly (p< 0.0001)**
